# Scene-Selectivity and Retinotopy in Medial Parietal Cortex

**DOI:** 10.3389/fnhum.2016.00412

**Published:** 2016-08-18

**Authors:** Edward H. Silson, Adam D. Steel, Chris I. Baker

**Affiliations:** ^1^Laboratory of Brain and Cognition, National institute of Mental HealthBethesda, MD, USA; ^2^Physiological Neuroimaging Group FMRIB Centre, John Radcliffe Hospital, University of OxfordOxford, UK

**Keywords:** retinotopy, population receptive fields, scene-selectivity, resting-state functional connectivity, memory

## Abstract

Functional imaging studies in human reliably identify a trio of scene-selective regions, one on each of the lateral [occipital place area (OPA)], ventral [parahippocampal place area (PPA)], and medial [retrosplenial complex (RSC)] cortical surfaces. Recently, we demonstrated differential retinotopic biases for the contralateral lower and upper visual fields within OPA and PPA, respectively. Here, using functional magnetic resonance imaging, we combine detailed mapping of both population receptive fields (pRF) and category-selectivity, with independently acquired resting-state functional connectivity analyses, to examine scene and retinotopic processing within medial parietal cortex. We identified a medial scene-selective region, which was contained largely within the posterior and ventral bank of the parieto-occipital sulcus (POS). While this region is typically referred to as RSC, the spatial extent of our scene-selective region typically did not extend into retrosplenial cortex, and thus we adopt the term medial place area (MPA) to refer to this visually defined scene-selective region. Intriguingly MPA co-localized with a region identified solely on the basis of retinotopic sensitivity using pRF analyses. We found that MPA demonstrates a significant contralateral visual field bias, coupled with large pRF sizes. Unlike OPA and PPA, MPA did not show a consistent bias to a single visual quadrant. MPA also co-localized with a region identified by strong differential functional connectivity with PPA and the human face-selective fusiform face area (FFA), commensurate with its functional selectivity. Functional connectivity with OPA was much weaker than with PPA, and similar to that with face-selective occipital face area (OFA), suggesting a closer link with ventral than lateral cortex. Consistent with prior research, we also observed differential functional connectivity in medial parietal cortex for anterior over posterior PPA, as well as a region on the lateral surface, the caudal inferior parietal lobule (cIPL). However, the differential connectivity in medial parietal cortex was found principally anterior of MPA. We suggest that there is posterior–anterior gradient within medial parietal cortex, with posterior regions in the POS showing retinotopically based scene-selectivity and more anterior regions showing connectivity that may be more reflective of abstract, navigationally pertinent and possibly mnemonic representations.

## Introduction

Functional magnetic resonance imaging (fMRI) studies investigating scene-perception in human reliably and repeatedly identify a trio of spatially distinct scene-selective regions, one on each of the lateral [occipital place area (OPA) or transverse occipital sulcus (TOS)] (Kravitz et al., 2011; Nasr et al., 2011; Dilks et al., 2013; Silson et al., 2015), ventral [parahippocampal place area (PPA)] (Epstein and Kanwisher, 1998) and medial [retrosplenial complex (RSC)] (Epstein, 2005, 2008; Aminoff et al., 2007) cortical surfaces.

Despite their shared category-selectivity, a division of labor between these regions has been suggested recently, with OPA and PPA reportedly more involved in the visual representation of scenes (MacEvoy and Epstein, 2007; Silson et al., 2015) and landmark coding (Marchette et al., 2015), respectively, whereas RSC is implicated in spatial memory (MacEvoy and Epstein, 2007; Marchette et al., 2015), navigation (Epstein and Higgins, 2007), topographical orientation (Kim et al., 2015), remembering previous events (Gilmore et al., 2016) and the anchoring of internal spatial representations (Marchette et al., 2014).

Recently, the presence and influence of retinotopic information has been demonstrated within both OPA (Nasr et al., 2011; Silson et al., 2015, 2016) and PPA (Arcaro et al., 2009; Silson et al., 2015). For example, we demonstrated differential representations of the contralateral lower and upper visual fields within OPA and PPA, respectively (Silson et al., 2015). These data suggest not only that computations within both OPA and PPA are likely constrained by their underlying visual field representations, but also, that each area is likely adapted to visual features that occur in the lower and upper visual fields, respectively (Silson et al., 2015). The magnitude of these biases, coupled with the consistency across individuals compelled us to ask whether RSC similarly exhibits systematic retinotopic biases. Unlike OPA and PPA, previous work provides conflicting evidence for the presence of retinotopic information within RSC. Although larger response magnitudes were reported for scenes presented contralaterally, fMRI adaptation was found not to depend on visual field position, indicating perhaps, the presence of receptive fields that span the vertical meridian (MacEvoy and Epstein, 2007) and suggesting RSC plays a more prominent role in spatial memory rather than scene perception *per se* (MacEvoy and Epstein, 2007). Others have reported a strong representation of the far periphery of the contralateral visual field within a similarly located region of medial parietal cortex identified through mental navigation (Huang and Sereno, 2013).

Here, we combined detailed mapping of both population receptive fields (pRF) and scene-selectivity with independently acquired resting-state functional connectivity analyses to explore scene and retinotopic processing in medial parietal cortex. Across participants, medial scene-selectivity was identified reliably within the posterior bank of ventral parieto-occipital sulcus (POS) and did not extend into retrosplenial cortex (Morris et al., 2000; Maguire, 2001; Epstein, 2008). We propose referring to this visually defined scene-selective region as the medial place area (MPA). Our analyses of pRF responses demonstrates a high degree of retinotopic sensitivity within MPA. Both left and right MPA exhibited significant biases for the contralateral visual field coupled with large receptive field sizes. Further, we confirmed this preference for contralateral space in MPA in an independent event-related quadrant experiment. Beyond its retinotopic sensitivity, our resting-state analyses highlight that MPA is more closely associated with the ventral than lateral surface. Dividing PPA into posterior and anterior subdivisions revealed a region of high differential connectivity, adjacent but anterior of scene- and retinotopically selective MPA. Taken together, these data suggest a posterior–anterior gradient of representation exists within medial parietal cortex, whereby the posterior bank of POS represents scene information retinotopically and anterior portions of medial parietal cortex mediate more spatial, navigational, and potentially mnemonic processes.

## Materials and Methods

### Participants

Sixteen participants (nine male, mean age = 31 years) completed retinotopic mapping (eight runs) and functional localizer (two runs) scans. Ten of these participants (six female, mean age = 28 years) also completed an additional event-related experiment with scenes presented in the four quadrants of the visual field. A separate group of 48 participants (33 female, mean age = 25 years) completed resting-state functional connectivity sessions. All participants had normal or corrected-to-normal vision and gave written informed consent in accordance with the declaration of Helsinki. The National Institutes of Health Institutional Review Board approved the consent and protocol. This work was supported by the Intramural Research program of the National Institutes of Health – National Institute of Mental Health Clinical Study Protocol 93-M-0170, NCT00001360.

### fMRI Retinotopic Mapping, Functional Localizer and Event-Related Sessions

Participants were scanned on either a research-dedicated GE 3 Tesla Signa scanner or a research-dedicated Siemens 7 Tesla Magnetom scanner in the Clinical Research Centre on the National Institutes of Health campus (Bethesda, MD, USA). Across scanners, oblique slices were oriented approximately parallel to the base of the temporal lobe and extended posteriorly through all of visual cortex. All participants completed at least eight runs of pRF mapping as well as two runs of an additional category-selective functional localizer. Participants in the event-related sessions completed six event-related runs as well as two runs of an additional category-selective functional localizer.

### 3T Scanning Parameters

Partial volumes of the occipital and temporal cortices were acquired using an eight-channel head coil (21 slices; 2 mm × 2 mm × 2 mm; 10% interslice gap; TR 2 s; TE 30 ms; matrix size, 96 × 96; FOV 192 mm).

### 7T Scanning Parameters

Partial volumes of the occipital and temporal cortices were acquired using a 32-channel head coil (42 slices; 1.2 mm × 1.2 mm × 1.2 mm; 10% interslice gap; TR 2 s, TE 27 ms; matrix size, 170 × 170; FOV 192 mm).

### Visual Stimuli and Tasks

#### Population Receptive Field Mapping

Population receptive field mapping sessions were conducted at either 3T (11 participants) or 7T (5 participants) field strengths. During pRF mapping sessions a bar aperture traversed gradually through the visual field, whilst revealing random scene fragments. During each 36 s sweep the aperture made 18 evenly spaced steps, one every 2 s (1TR), to traverse the entire screen. During each bar position (1TR) five scene fragments were displayed in rapid succession (400 ms per image). Across the 18 aperture positions all 90 possible scene images were displayed once. Thus, in any single sweep, each scene occurred only once, reducing the likelihood that participants mentally ‘fill-in’ in the underlying image, a problem that can arise if a single background image is revealed gradually. A total of eight sweeps were performed in each run (four orientations, two directions). Specifically, the bar aperture progressed in the following order for all (8) runs: Left–Right, Bottom Right–Top Left, Top–Bottom, Bottom Left–Top Right, Right–Left, Top Left–Bottom Right, Bottom–Top, and Top Right–Bottom Left. The bar stimuli covered a circular aperture (20° diameter 7T; 15° diameter 3T. During runs, participants performed a color detection task at fixation, indicating via button press when the white fixation dot changed to red. Color fixation changes occurred semi-randomly, with approximately two color changes per sweep.

#### Category-Selective Functional Localizers

Functional localizer runs were conducted at either 3T (11 participants) or 7T (5 participants) field strengths. All participants completed two runs in order to localize scene- and face-selective areas. These scans employed an on/off design (scenes/faces) with 16 alternating blocks (16 s) of 20 stimuli (5° × 5°) presented while participants performed a one-back task. Within a block, 20 stimuli were presented for 300 ms each, separated by a 500 ms inter-stimulus interval. Additionally five participants completed independent localizer scans employing a multiblocked design. In these sessions, images from eight categories including faces, buildings, scenes (manmade/natural, open/closed), and man-made and natural objects, were presented in 16-s blocks with an 8 s blank fixation period separating blocks. Each category was presented twice per run, and the order of presentation was counterbalanced across participants and runs.

#### Event-Related Scene Quadrant Presentation

Event-related scanning sessions were conducted at either 3T (six participants) or 7T (four participants) field strengths. Participants performed an attention-demanding task at fixation whilst whole-scene images were presented randomly in one of the four quadrants of the visual field (top left, top right, bottom left, and bottom right). Stimuli subtended 6.5° × 6.5° and were centered 6.5° from the central fixation cross into one of the quadrants of the visual field. Participants maintained fixation throughout. Scene stimuli were scaled to subtend the same visual angle of the screen during both 3T and 7T scans. As a scene was presented, one arm of the fixation cross, either the horizontal or the vertical, increased in length. Participants were required to identify, via button response, the longer fixation arm. Stimulus presentation and fixation cross changes occurred simultaneously. Within each run, each scene (*n* = 24) appeared at each location (*n* = 4) for 400 ms, with a jittered (4–12 s) interstimulus fixation period; thus, each run contained 96 trials. The order of presentations and fixation arm extensions was randomized within each run. Participants completed six runs of the event-related experiment.

Population receptive field and functional localizer fMRI preprocessing. All data were analyzed using the Analysis of Functional NeuroImages (AFNI) software package^1^ (Cox, 1996). All functions and programs are readily available in the current version: AFNI binary version February 10, 2016. Prior to functional localizer, event-related and pRF analyses, all images for each participant were motion corrected to the first volume of the first run, after removal of the appropriate ‘dummy’ volumes (8) to allow stabilization of magnetization. Post motion-correction data were smoothed with a 2 mm full-width at half-maximum Gaussian kernel for both 3T and 7T localizer runs and event-related runs.

### Localizer Analysis

To identify scene- and face-selective regions of interest (ROIs), significance maps of the brain were computed in each participant by performing a correlation analysis between the assumed hemodynamic response function and the activation time courses thresholded at *p* < 0.0001 (uncorrected). Significance maps were then projected onto surface reconstructions of the gray–white matter boundary of individual hemispheres. Only contiguous clusters of nodes exceeding the above threshold were defined as scene- or face-selective. The anatomical locations of these clusters were then inspected in order to define ROIs consistent with previously published work (Sayres and Grill-Spector, 2008; Schwarzlose et al., 2008; Kravitz et al., 2011; Nasr et al., 2011; Marchette et al., 2014, 2015; Silson et al., 2015).

### Event-Related Analysis

For event-related runs, performing *t*-tests between each condition (top left, bottom left, top right, and bottom right) and baseline generated significance maps. The β-values for each condition were extracted from voxels within each ROI and averaged.

### Population Receptive Fields Mapping Analysis

Population Receptive Field analyses of unsmoothed data were conducted in AFNI, using a pRF implementation for the AFNI distribution (Silson et al., 2015). The model divides the stimulated field of view into an X, Y grid with 200 samples across its height and width, and for each position in that X, Y grid, sigma (pRF size) values are simultaneously sampled at the same resolution, but over a default range of 0 to half the size of the field of view (sampled at 100 even intervals). These default parameters result in 4 million possible pRF’s (all possible combinations of X, Y location and sigma). Given the position of the stimulus in the visual field at every TR, the estimated time series for a pRF of a given location (X, Y) and size (sigma) can be estimated by convolving these estimates with a 2-D stimulus time series, which contains binary masks of the stimulus location at each TR. Both Simplex and Powell optimization algorithms are utilized to find the best time series/parameter sets (X, Y, and sigma) by minimizing the least-squares error of the predicted time series measured against the acquired time series in each voxel. The model outputs for each voxel the X, Y location representing the center of the receptive field; sigma, which represents the diameter (size) of the receptive field; and *R*^2^, which corresponds to the explained variance of the fit and can be used to statistically threshold these data.

Visual field coverage plots, which are built by combining the best Gaussian receptive field model for each voxel within an ROI, can be computed from these data. The coverage plots for a given ROI are an aggregation of these Gaussians. Assuming a strong central tendency in the centers of the receptive fields, a linear aggregation (e.g., summation) will result in a coverage plot appearing as a single large Gaussian. In our analyses a max operator is used. This creates a coverage plot that reflects, at each point, the maximum pRF value from all of the receptive field models within an ROI (Winawer et al., 2010). Thus, the coverage plot reflects the maximum envelope of all the Gaussians within an ROI. While this allows for a non-symmetric shape, the edges of that plot will often evidence a Gaussian falloff as few pRFs define the edges.

### Delineation of Visual Field Maps

To rule out the possibility that our MPA ROI was simply an extension of retinotopy present within antecedent visual areas, we identified V1, V2d, V2v, V3d, V3v, and hV4 in all participants. To identify visual field maps in individual participants, the representations of polar angle and eccentricity were visualized on surface reconstructions of both hemispheres and inspected. Surface reconstructions of the gray and white matter boundary of individual participant hemispheres were made using the Freesurfer4 autorecon script^2^. Retinotopically organized maps were visible and present in all tested hemispheres. Notwithstanding subtle inter-participant variability, the main features of the maps, in particular the reversals in visual field representation at the vertical and horizontal meridians were consistent across participants. In accordance with previous reports (Engel et al., 1994; Sereno et al., 1995; DeYoe et al., 1996; Larsson and Heeger, 2006; Wandell et al., 2007) retinotopic visual field maps were delineated using the following criteria: (1) the polar angle representations displayed reversals. That is, the representations of polar angle in neighboring visual areas were mirror reversals of one another, with a reversal in the representation along their shared boundary; (2) the polar angle and eccentricity components within each visual area were organized largely orthogonal to one another.

#### Resting-State Functional Connectivity

##### 3T scanning parameters

Volumes of the whole brain were acquired using a 32-channel head coil. Multi-echo EPI scans were collected with the following parameters: TEs = 14.9, 28.4, 41.9 ms, TR = 2 s, ASSET acceleration factor = 2, flip-angle = 65°, bandwidth = 250.000 kHz, FOV = 24 cm × 24 cm, acquisition matrix = 64 × 64, resolution = 3.4 mm × 3.4 mm × 3.4 mm, slice gap = 0.3 mm, 34 slices per volume covering the whole brain. Respiratory and cardiac traces were recorded. For resting-state MRI runs, the first 30 volumes were discarded. In each participant two independent 20-min rest periods (eyes closed) were acquired.

##### Resting state fMRI preprocessing

Preprocessing of the fMRI data was performed using AFNI. Each echo was processed independently prior to optimal combination of the data from each TE (see below). Slice-time correction was applied (3dTShift) and signal outliers were attenuated [3dDespike (Jo et al., 2013)]. Motion correction parameters were estimated relative to the first volume of the middle TE (28.4 ms), and registered to the structural scan (3dSkullStrip, 3dAllineate). These registration parameters were then applied in one step (3dAllineate) and the data were resampled to 3 mm isotropic resolution. The three TEs were combined as described below.

The optimal echo time for imaging the BOLD effect is where the TE is equal to T2^∗^. Because T2^∗^ varies across the brain, single echo images are not optimal to see this variation. By acquiring multiple echoes, this enables the calculation of the “optimal” T2^∗^ weighted average of the echoes, which allows one to recover signals in dropout areas and improves contrast-to-noise ratio (Posse et al., 1999; Poser et al., 2006; Kundu et al., 2014; Evans et al., 2015).

After optimal combination, we applied the basic ANATICOR (Jo et al., 2010) procedure to yield nuisance time series for the ventricles and local estimates of the BOLD signal in white matter. All nuisance time-time series [six parameters of motion, local white matter, ventricle signal, and six physiological noise regressors (AFNI: RetroTS)] were detrended with fourth order polynomials. For rest data, these regressors, along with a series of sine and cosine functions to remove all frequencies outside the range (0.01–0.25 Hz) were regressed out in a single regression step (AFNI program 3dTproject).

Time points with motion greater than 0.3 mm were removed from the data [scrubbing, see Power et al. (2012)] and replaced with values obtained via linear interpolation in time. These cleaned time series were aligned to the standard mesh for subsequent analysis (@Suma_AligntoExperiment). No smoothing was applied to these data.

##### Resting state analysis

Category-selective ROIs for resting state analysis were derived from the group data (*n* = 16). Using the contrasts described above, these ROIs [PPA, OPA, MPA, fusiform face area (FFA), and Occipital face area (OFA)] were restricted to the top 300 category selective nodes in each region to account for large differences in ROI size. This thresholding procedure revealed anterior and posterior components of PPA, which were considered separately (aPPA and pPPA) in some analyses, resulting in the following ROI set (MPA, OPA, pPPA, aPPA, FFA, and OFA).

## Results

### Scene-Selectivity in Medial Parietal Cortex

**FIGURE 1:**
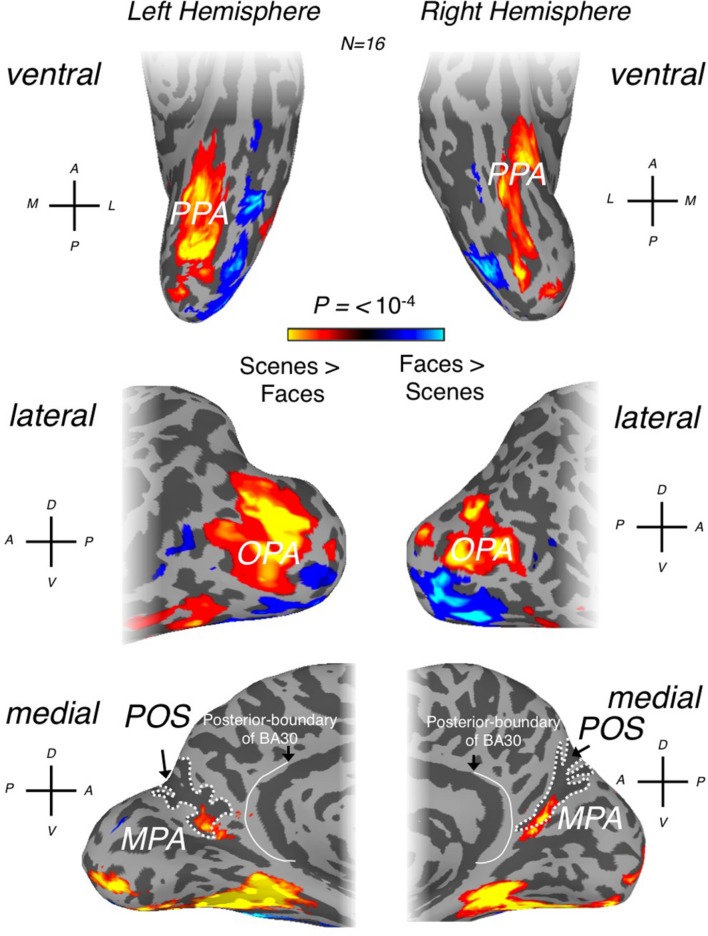
**Scene- and face-selective regions of occipitotemporal and medial parietal cortex**. Group averaged (*n* = 16) responses to scenes > faces (*p* < 0.0001) are overlaid in false colors on surface reconstructions of the left (left column) and right (right column) hemispheres of a sample participant. Regions responding significantly more to scenes are overlaid in hot colors with regions responding significantly more to faces overlaid in cold colors. (Top row) Ventral surface views demonstrate that parahippocampal place area (PPA) is readily identifiable in both hemispheres, located medially of face-selective responses (see orientation labels inset: A, anterior; P, posterior; M, medial; L, lateral). The location of PPA is consistent with a large number of previous studies. (Middle row) occipital place area (OPA) can be seen on lateral surface views of both hemispheres. OPA can be seen to cover a relatively large swath of cortex on the lateral surface and is located superior to face-selective responses. (Bottom row) medial place area [MPA; or retrosplenial complex (RSC)] can be seen within the posterior and ventral banks of the parieto-occipital sulcus (POS; white-dashed line defines the outer edges of POS) on medial surface views of both hemispheres. The posterior boundary of Brodmann area 30 (BA) is also shown on both hemispheres, redrawn from Brodmann (1909) and Vann et al. (2009).

Initially, scene-selective regions, one on each of the lateral (OPA), ventral (PPA), and medial (RSC) cortical surfaces were identified, where possible, in each participant (OPA = 16 bilaterally, PPA = 16 bilaterally, RSC = 13 left hemisphere and RSC = 15, right hemisphere) using the contrast of scenes > faces (*p* < 10^-4^, uncorrected). The locations of our OPA, PPA and RSC ROIs (**Figure 1**) are entirely consistent with previous literature (Epstein and Kanwisher, 1998; Epstein, 2005, 2008; Kravitz et al., 2011; Nasr et al., 2011; Baldassano et al., 2013; Silson et al., 2015, 2016).

As others have noted (Nasr et al., 2011), despite the commonly used name, ‘RSC,’ the scene-selective activation in medial parietal cortex appears to lie largely outside retrosplenial cortex proper. Indeed, RSC is identified frequently within the posterior bank of ventral POS, in close proximity to, but importantly spatially distinct from, the peripheral representations of both primary visual cortex (V1) and V2d (Epstein, 2005, 2008; Nasr et al., 2011; Baldassano et al., 2013; Stevens et al., 2015). However, human retrosplenial cortex is located largely within the callosal sulcus, extending onto the cingulate gyrus primarily posteroventrally, specifically the isthmus of the cingulate gyrus (Morris et al., 2000; Maguire, 2001; Epstein, 2008). Given that the peak of the scene-selective activation is largely contained within the POS and that at a standard threshold (for example see Sayres and Grill-Spector, 2008; Kravitz et al., 2010, 2011) the group activation does not extend onto the cingulate gyrus, we think that the continued use of ‘retrosplenial’ to refer to this region is potentially misleading. Instead, we suggest referring to this visually defined scene-selective region as the medial place area (MPA).

**FIGURE 2:**
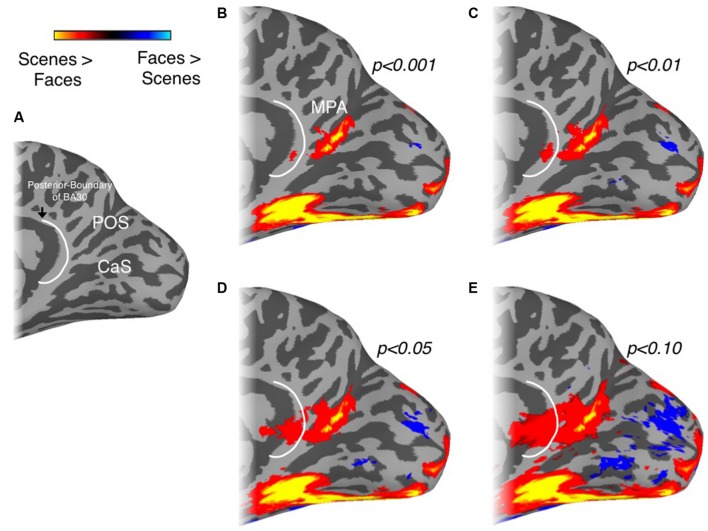
**Localization of MPA as a function of statistical threshold. (A)** Medial view of the right hemisphere of a representative participant is shown with the POS and calcarine sulcus (CaS) labeled. The posterior boundary of BA30, redrawn from Brodmann (1909) and Vann et al. (2009) is also overlaid (white-line). The spatial extent of our group-based MPA regions of interest (ROI) with respect to the posterior-boundary of BA30 at different thresholds is demonstrated in **(B–E)**. At lower thresholds, a small peak of activation does emerge on the cingulate gyrus, potentially corresponding to BA29/30, but it is dorsal to the isthmus and only extends into the callosal sulcus, where most of retrosplenial cortex is contained, at very low thresholds.

To determine to what extent this localization of scene-selectivity outside of retrosplenial cortex is dependent on the statistical threshold we employed, we systematically varied this threshold (**Figure 2**). At lower thresholds, a small peak of activation does emerge on the cingulate gyrus, potentially corresponding to BA29/30, but it is dorsal to the isthmus and only extends into the callosal sulcus, where most of retrosplenial cortex is contained, at very low thresholds (**Figures 2D,E**). To investigate individual variability in the spatial extent of the MPA, we calculated in how many participants the scene-selective activation extended onto the cingulate gyrus and into the commonly drawn BA 29/30. In the left hemisphere, activation in BA29/30 was only present in 2/13 participants and in 1/15 participants in the right hemisphere. Finally, to investigate to what extent the specific contrast we employed (scenes vs. faces) impacts the spatial extent of the medial parietal scene-selectivity, we compared the extent of scene-selective activation in a subset of our participants (*n* = 5) who participated in a separate experiment allowing us to define scene-selective activation by the contrast scenes vs. objects. This alternative contrast identified a similarly located region within the POS (see Supplementary Figure S1). Importantly, in all participants the spatial extent of the ROI based on scene vs. objects did not extend anteroventrally in the direction of restrosplenial cortex beyond the boundary defined by scenes vs. faces.

### Retinotopic Profile of MPA

Historically, scene-selective regions in medial parietal cortex have been implicated in the processing and mediating of relatively high-level visual and spatial processes such as spatial memory (MacEvoy and Epstein, 2007; Marchette et al., 2015), navigation (Epstein and Higgins, 2007), topographical orientation (Kim et al., 2015), remembering past events (Gilmore et al., 2016), and the anchoring of internal spatial representations (Marchette et al., 2014), rather than thought of as sensitive to visual field position, as would be predicted of a retinotopically responsive region.

**FIGURE 3:**
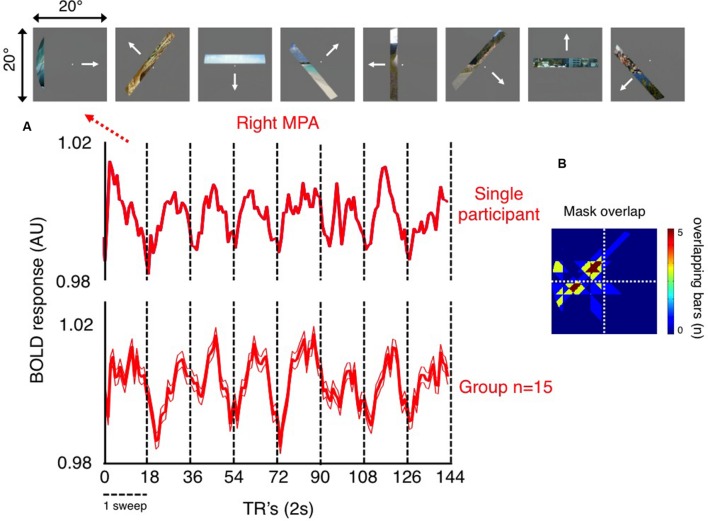
**Retinotopic sensitivity in right MPA. (A)** The average time-series during pRF mapping of all voxels within the right MPA of a single participant (top time-series), and the group-average (bottom time-series; ±standard error of the mean, SEM) are shown. These time-series show clearly eight peaks of activity. In both examples, each peak occurs once during each of eight sweeps of our mapping stimulus (18 TR’s per sweep; vertical dashed black lines denote the start/end of each sweep). **(B)** The portion of the visual field at which bar positions corresponding to each peak (corrected for the delay in hemodynamic response) of the group-averaged time-series overlap is also shown. These bars overlap largely within the left (contralateral) visual field. This plot represents the portion of the visual field that right MPA is most sensitive to.

To explore the retinotopic profile of MPA, we initially calculated, in each participant, the mean time-series of all voxels within both left and right MPA and then averaged across participants. Notwithstanding subtle differences in the pRF’s across voxels, which will exhibit different time-courses, the existence of retinotopically sensitive responses following such averaging demonstrates the retinotopic sensitivity of this region at a gross level. These time-courses are shown for the right hemisphere of a representative participant and at the group level in **Figure 3**.

Inspection of **Figure 3** reveals a number of interesting features. First, the fact that MPA voxels exhibit sensitivity to our simple and restricted stimuli (bar containing a restricted fragment of a scene), shows that scene features, without detailed context, are sufficient to drive this region. Second, in both single participant and group time-series, eight peaks of activity are clearly evident, which reflect the eight sweeps our pRF mapping stimulus made through the visual field and thus through the pRF’s of MPA voxels (**Figure 3A**). Third, the stimuli at the top of **Figure 3A**, shows position in the visual field corresponding to the peak of our group level time-series during each sweep (adjusted temporally to account for the lag in hemodynamic response). The region of visual space over which these bar positions overlap maximally, reflecting the positions in the visual field that right MPA is most sensitive to are shown in (**Figure 3B**). Importantly, although this region covers portions of the visual field both above and below the horizontal meridian, they are restricted exclusively to the left (contralateral) visual field. Finally, the width of each peak of activity is wide, spanning much of the width of each sweep (dashed vertical lines in **Figure 3A**), which is suggestive of large receptive fields within MPA (although it is also possible that this reflects a population of neurons with small receptive fields with limited spatial overlap). Crucially, if MPA were retinotopically insensitive, such a systematic pattern of activity would be unlikely to occur.

**FIGURE 4:**
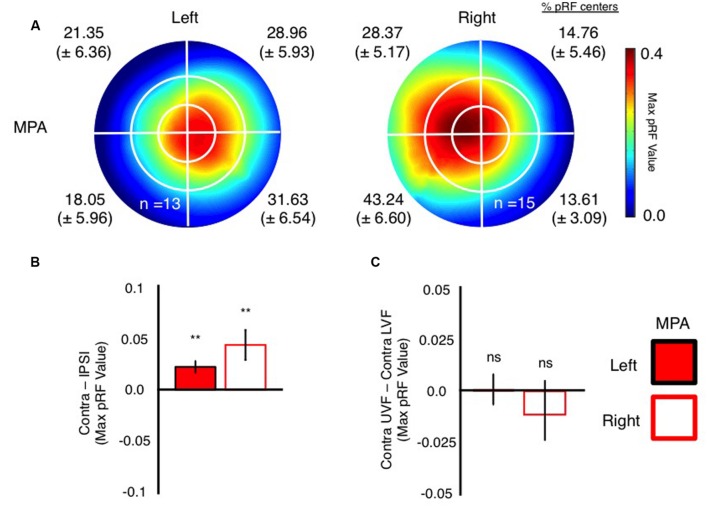
**Visual field coverage and visual field biases in left and right MPA. (A)** Group-average visual field coverage for the left and right MPA are shown. Both ROIs exhibit a bias for the contralateral visual field, coupled with large receptive fields. The percentage of pRF centers (Mean ± SEM) within each quadrant of the visual field is given for each ROI and confirms the contralateral visual field bias in MPA bilaterally. **(B)** Bars depict the contralateral biases present in left and right MPA. In both ROIs, bars depict the mean value in the contralateral *minus* ipsilateral visual fields. Contralateral biases were found to be significant (relative to zero) in both ROIs (^∗∗^*p* < 0.01). **(C)** Bars depict the elevation bias present within left and right MPA. Bars depict the pRF value in the contralateral upper *minus* contralateral lower visual fields. Elevation biases were not significant in either ROI.

Next, we sought to characterize MPA’s receptive field profile in a number of ways that mirror our previous characterizations of retinotopy within both OPA and PPA (Silson et al., 2015, 2016). Initially, visual field coverage plots (Winawer et al., 2010; Silson et al., 2015) were computed for each participant and then averaged across participants (**Figure 4**). These plots were computed from pRF values derived from all nodes within the functionally localized MPA ROIs, without thresholding of the pRF values themselves. Both left and right MPA exhibit clear biases for the contralateral visual field, with more pRF’s centered within, and more representation of, the contralateral visual field in both hemispheres (see proportion of pRF centers per visual quadrant inset in **Figure 4A**). Such contralaterally biased representations mirror those present in both left and right OPA and PPA, respectively (MacEvoy and Epstein, 2007; Arcaro et al., 2009; Kravitz et al., 2013; Silson et al., 2015). Unlike its lateral and ventral counterparts, however, MPA does not exhibit a consistently clear bias for a single visual quadrant in either hemisphere. Indeed, representations of the contralateral visual field were largely equivalent about the horizontal meridian in both regions (**Figure 4A**). We tested for any biases for a single quadrant of the visual field using repeated-measures ANOVA with factors Hemisphere (Left, Right), Visual Field (Ipsilateral, Contralateral) and Vertical Position (Upper, Lower). There was a main effect of Hemisphere [*F*_(1,13)_ = 18.17, *p* < 0.001], reflecting on average stronger responses in the right than left hemisphere. The main effect of Visual Field was also significant [*F*_(1,13)_ = 10.45, *p* < 0.007], reflecting on average stronger responses in the contralateral visual field within both regions. Further, there was a significant Hemisphere by Visual Field interaction [*F*_(1,13)_ = 4.72, *p* < 0.49], reflecting on average a stronger contralateral bias in the right than left hemisphere. However, there was no main effect of Vertical Position [*F*_(1,13)_ = 0.791, *p* = 0.39] and no interactions involving Vertical Position reflecting on average the largely equal representations within the upper and lower visual fields within both regions. These data demonstrate that both left and right MPA exhibit a contralateral bias, but no bias for a specific quadrant of the visual field.

To further quantify these biases, we calculated contralateral (contralateral *minus* ipsilateral pRF value) and elevation (contralateral upper *minus* contralateral lower pRF value) bias measurements in each participant and ROI. Consistent with our previous characterizations of OPA and PPA (Silson et al., 2015), MPA exhibited significant contralateral biases (*p* < 0.05, relative to a zero bias assumption) in both hemispheres (**Figure 4B**). In contrast however, the elevation biases within MPA were not significant in either hemisphere (**Figure 4C**).

**FIGURE 5:**
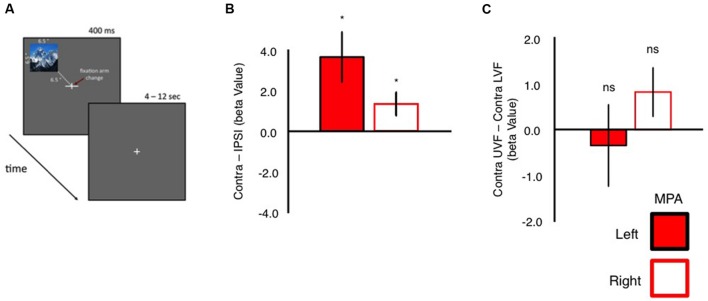
**Event-related quadrant design and univariate analyses. (A)** Schematic representation of our event-related quadrant experiment. Twenty-four scene images were presented randomly (400 ms) into one of the four quadrants of the visual field. Each image was centered 6.5° into each visual field quadrant. Participants performed an orthogonal task at fixation. During presentation of scene stimuli, one arm of the fixation cross (horizontal or vertical) became elongated. Participants indicated via button press which arm was elongated. **(B)** Group-average contralateral biases in left and right MPA. Bars reflect the beta values for stimuli presented in the contralateral *minus* ipsilateral visual field. Contralateral responses were significant (relative to zero) in both left and right MPA. **(C)** Group-average elevation biases in left and right MPA. Bars depict the beta values for stimuli presented in the contralateral upper *minus* contralateral lower visual field. Elevation biases were not significant (relative to zero) in either left or right MPA (^∗^*p* < 0.05).

To confirm these retinotopic biases within MPA, independently, we measured responses in MPA during a condition-rich event-related experiment. Participants viewed 24 whole scene images presented randomly into one of the four quadrants of the visual field, whilst performing an orthogonal task at fixation (**Figure 5A**). In agreement with our pRF mapping, univariate analyses (**Figure 5B**) demonstrated that both left and right MPA exhibited stronger responses for stimuli presented in the contralateral over ipsilateral visual fields (see Supplementary Figure S2 for average β-values for all conditions collapsed across hemispheres). Again contralateral and elevation bias measurements (computed as above) were calculated for each participant and region. Both left and right MPA exhibited significantly stronger responses (*p* < 0.05, relative to zero bias assumption) to stimuli in the contralateral visual field, confirming this organizational principle within MPA. Elevation biases in MPA, however, were not significant for either hemisphere (**Figure 5C**).

**FIGURE 6:**
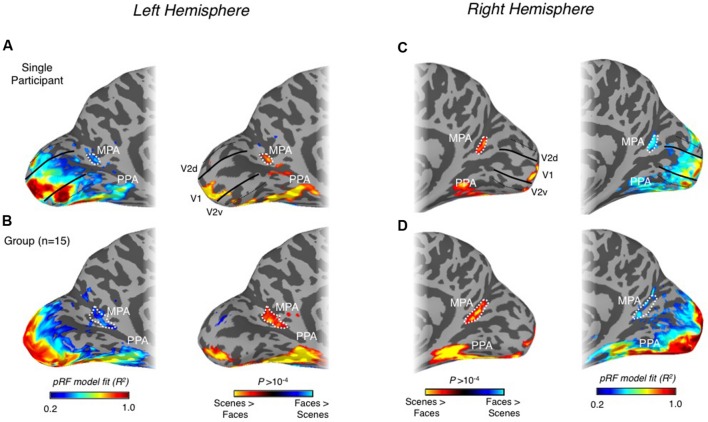
**Spatial correspondence between scene-selectivity and retinotopy in medial parietal cortex. (A)** Retinotopic responses (left column) and scene-selectivity (right column) are shown on medial surface views of the left hemisphere for a single participant. A region showing strong retinotopic responses can be seen largely overlapping the MPA ROI (white-dashed line). The boundaries of V1 (solid black lines) and the boundaries between V2v and V3v and between V2d and V3d (dashed black lines) are also shown. **(B)** Group-average retinotopic responses and scene-selectivity are shown on the same surface reconstruction as in **(A)**. Strong retinotopic responses can be seen to overlap with the group-defined MPA. **(C)** Retinotopic responses (left column) and scene-selectivity (right column) are shown on medial surface views of the right hemisphere for a single participant. A region showing strong retinotopic responses can be seen overlapping the MPA ROI almost entirely. **(D)** Group-average retinotopic responses and scene-selectivity are shown on the same surface reconstruction as in **(A)**. Strong retinotopic responses can be seen to overlap with the group-defined MPA.

Finally, we examined whether MPA could be identified based solely on retinotopy. In particular, we asked whether the quality of our pRF estimates (explained variance of the pRF model) could localize this region. Importantly, across participants we observed reliable and statistically significant pRF responses within the posterior and ventral bank of POS in both hemispheres (**Figure 6**). Indeed, this region either encompassed entirely or overlapped substantially with our MPA ROIs in many participants, but importantly, was separated spatially from both antecedent visual areas (V1 and V2d) and retinotopic maps found in more dorsal regions of POS, such as V6, V6Ad, and V6Av (Pitzalis et al., 2006, 2015; Cardin et al., 2012). The spatial overlap between scene-selective MPA and retinotopic sensitivity in both hemispheres is shown for a representative participant and at the group level in **Figure 6**.

Taken together, our characterization of MPA pRF’s suggests that, MPA represents predominantly the contralateral visual field. Unlike its lateral and ventral surface counterparts, however, MPA does not exhibit a consistent bias for a single quadrant of the visual field, but rather, exhibits an apparent full hemifield representation.

### Resting-State Reveals Differential Connectivity among Scene-Selective Regions

To explore further the relationship between MPA and scene-selective regions on both lateral (OPA) and ventral surfaces (PPA), we analyzed functional imaging data collected from a separate group of 48 participants whilst at rest.

**FIGURE 7:**
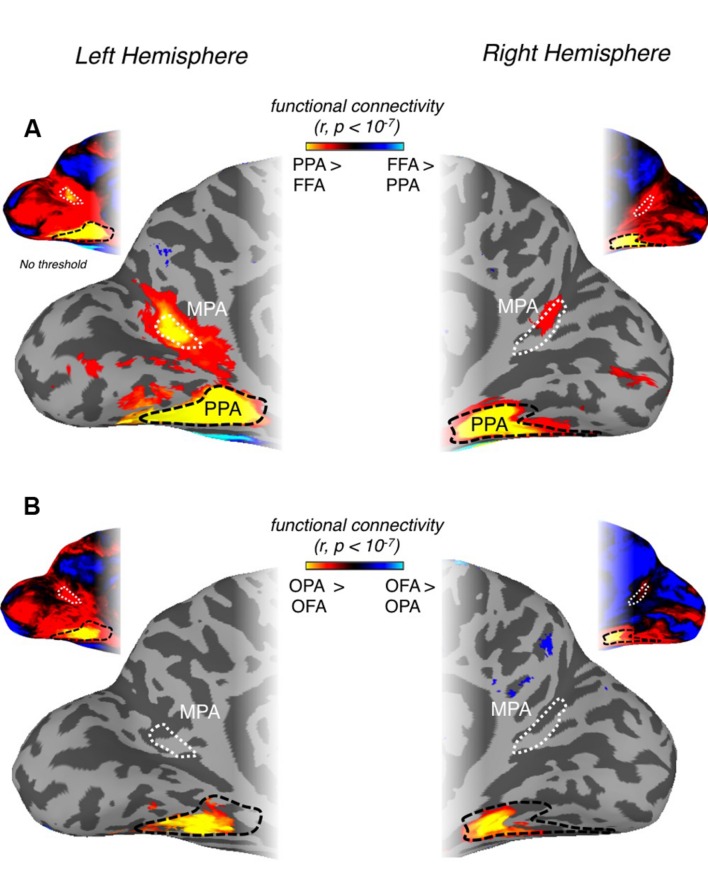
**Differential patterns of resting-state functional connectivity between scene- and face-selective regions on both ventral and lateral surfaces. (A)** Medial views of the left (left column) and right (right column) hemisphere of a representative participant are shown with the group-average differential connectivity maps for PPA vs. FFA (*ventral surface contrast)*. Unthresholded versions in both hemispheres are inset above. The group-level MPA (white-dashed line) and PPA (black-dashed line) ROIs are identified. A region within the POS, exhibiting significantly greater PPA connectivity, can be seen to overlap with our MPA ROI in both hemispheres. **(B)** The same region is not visible when the contrast of OPA vs. OFA is performed and thresholded at the same significance level (*p* < 10^-7^). Again unthresholded versions are inset above for both hemispheres.

Initially, we examined whether MPA could be identified on the basis of differential connectivity with scene- vs. face-selective regions on both the ventral and lateral surfaces, considered independently. Our principal localization of MPA was based on the direct contrast of scenes > faces, for consistency therefore, we computed differential connectivity measurements for (i) PPA vs. FFA (*ventral surface contrast*) and (ii) OPA vs. OFA (*lateral surface contrast*). A region overlapping our MPA ROI was clearly identifiable on the basis of differential connectivity between PPA and FFA in both hemispheres (**Figure 7A**), but not, between OPA and OFA (**Figure 7B**), suggesting that MPA is more strongly associated with PPA than OPA.

### Posterior–Anterior Gradient Within Medial Parietal Cortex

**FIGURE 8:**
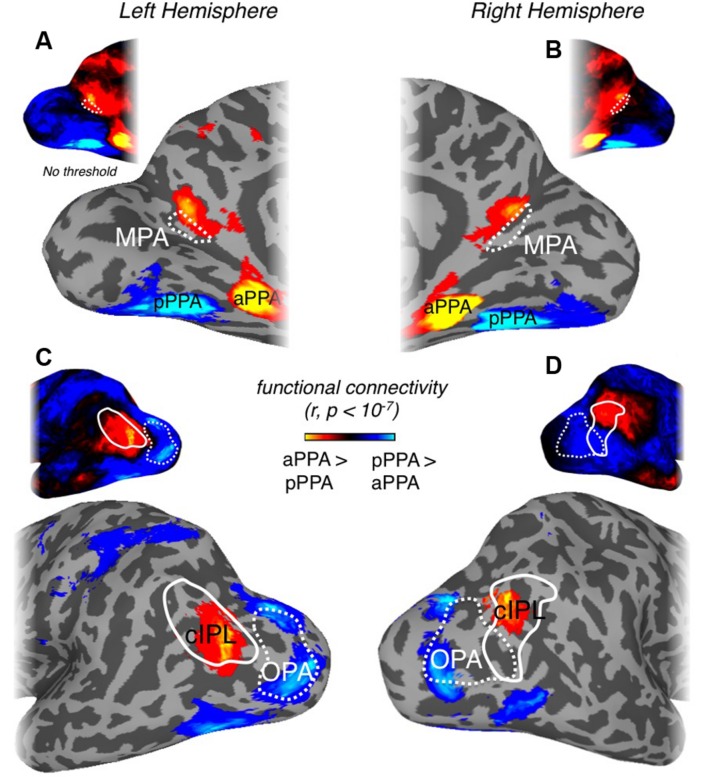
**Differential connectivity patterns between posterior and anterior PPA. (A)** Medial view of the left hemisphere of a representative participant is shown. Regions of cortex showing significant (*p* < 10^-7^) differential connectivity with anterior over posterior PPA (hot colors) or vice-versa (cold colors) at the group-level are overlaid. A region showing significant connectivity with anterior PPA can be seen directly adjacent and anterior to our scene- and retinotopically sensitive MPA ROI (white dashed-line), referred to as anterior MPA (aMPA). Although MPA does demonstrate stronger anterior PPA connectivity in the raw correlation (see unthresholded version inset above) this does not survive statistical analysis. **(B)** Medial view of the right hemisphere. A region showing significant anterior PPA connectivity can be seen largely anterior of our MPA ROI (unthresholded version inset above). **(C)** Lateral view of the left hemisphere of a representative participant. A region overlapping with the cIPL (white solid-line) showing significantly greater anterior PPA connectivity can be seen. Scene-selective OPA (white dashed-line) is more strongly correlated with posterior PPA. The unthresholded version is inset above. **(D)** Lateral view of the right hemisphere of a representative participant. A region overlaping with the cIPL (white solid-line) showing significantly greater anterior PPA connectivity can be seen. Scene-selective OPA (white dashed-line) is more strongly correlated with posterior PPA. The unthresholded version is inset above.

Previous work has suggested that medial scene-selective regions show stronger functional connectivity with anterior PPA (aPPA) than posterior PPA (pPPA; Baldassano et al., 2013). Given these prior results, we analyzed our resting-state data with respect to pPPA and aPPA (see Materials and Methods), by computing for each node, the differential connectivity between aPPA vs. pPPA. This differential connectivity analysis, illustrated in **Figure 8**, revealed a number of noteworthy features. First, significant differential aPPA-pPPA connectivity was found within medial parietal cortex, but was largely localized to a region directly adjacent and anterior of MPA in the anterior bank of ventral POS and extending into the precuneus in both hemispheres (**Figures 8A,B**). Second, highly differential aPPA-pPPA connectivity was also found on the lateral surface, overlapping considerably with the caudal inferior parietal lobule (cIPL) ROI, taken from the Eickhoff–Zilles PGP probabilistic cytoarchitectonic map (Eickhoff et al., 2005, based on Caspers et al., 2006, 2008; **Figures 8C,D**) consistent with prior work (Baldassano et al., 2013). In contrast, however, lateral portions of visual cortex including OPA and face-selective regions OFA and FFA were significantly more associated with pPPA than aPPA (**Figures 8C,D**). The spatial adjacency but limited overlap between scene-selective MPA in the posterior bank of POS and the more anterior precuneus region showing significant association with aPPA, suggests a posterior–anterior gradient exists within medial parietal cortex and the POS.

To investigate the functional connectivity in medial parietal cortex further, we created a connectivity-defined ROI (CON) based on the differential connectivity between aPPA vs. pPPA. Importantly, we defined this connectivity ROI on the basis of differential connectivity data acquired during one rest period and subsequently applied this ROI to independent data acquired during a separate rest period within each participant (see Materials and Methods).

**FIGURE 9:**
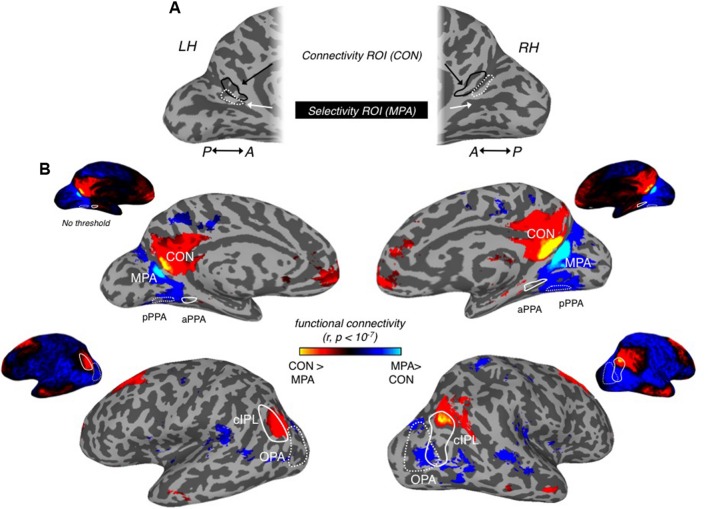
**Differential connectivity patterns between MPA and connectivity ROI (CON). (A)** Medial views of the left hemisphere (left) and right (right) hemisphere of a representative participant are shown. Scene-selective MPA can be seen within the posterior and ventral banks of the POS in both hemispheres (dashed-white line). Our CON can be seen more anteriorly within POS in both hemispheres (soild-black line). **(B)** Group-level differential connectivity patterns between MPA (cold colors) and CON (hot colors) are overlaid onto surface reconstructions of both hemispheres, with unthresholded versions inset above. Medial views are shown along the top-row, with lateral views shown along the bottom-row. (Top row) in both left (left column) and right (right column) medial views, significant (*p* < 10^-7^) aMPA connectivity can be seen extending into the precuneus and also within orbitofrontal regions, whereas significant MPA connectivity is seen largely posterior of MPA. Posterior PPA (dashed-white line) and anterior PPA (solid-white line) ROIs are also highlighted. (Bottom row) In both left (left column) and right (right column) lateral views, significant aMPA connectivity can be seen overlapping the cIPL (solid-white line), and within anterior temporal and superior frontal regions. Significant MPA connectivity is observed more posteriorly overlapping OPA.

Next, to compare the connectivity profiles of the selectivity-defined MPA with the more anterior CON, we directly contrasted the connectivity patterns for these two regions in both hemispheres (**Figure 9A**). Consistent with our pRF analyses, MPA exhibited stronger connectivity than CON with visual cortical areas, in particular pPPA and OPA, which have been shown previously to evidence strong retinotopy (Silson et al., 2015, 2016). In contrast, the CON ROI showed stronger functional connectivity than MPA with the precuneus (medially), aPPA (ventrally) and with the inferior parietal lobule (laterally) that overlapped with cIPL (**Figure 9B**). Stronger CON connectivity was also present within anterior temporal, as well as, orbitofrontal and superior frontal cortical locations (**Figure 9B**).

**FIGURE 10:**
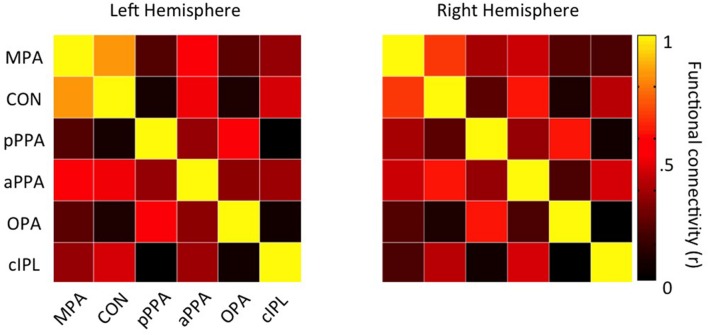
**Functional connectivity matrices for all scene-related ROIs**. Group-average (*n* = 48) connectivity matrices between all scene-related ROIs (MPA, CON, pPPA, aPPA, OPA, and cIPL) are shown for the left and right hemispheres, respectively.

To characterize the functional connectivity profiles of CON and MPA further, we focused on all scene-related ROIs (MPA, CON, pPPA, aPPA, OPA, and cIPL) and first computed all pair-wise time course correlations (**Figure 10**). In general, there are positive correlations between all these ROIs in both hemispheres. However, CON shows very low correlation with both pPPA and OPA (regions that are generally considered to represent the visual properties of scenes). Next we analyzed the relative strength of connectivity of MPA and CON by computing differential correlations between our medial parietal ROIs (MPA and CON) and scene-related regions on the (i) ventral surface (pPPA, aPPA), and (ii) lateral surface (OPA and cIPL) in both hemispheres. For each surface we conducted separate two-way repeated-measures ANOVAs with Hemisphere (Left, Right) and Seed ROI (MPA, CON).

**FIGURE 11:**
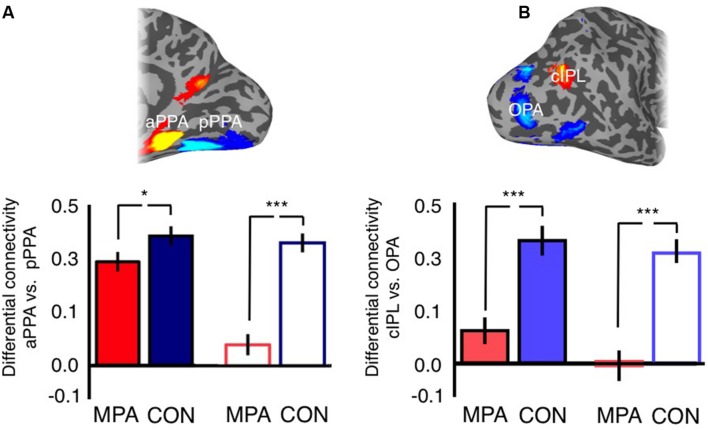
**Posterior–anterior gradient across medial parietal cortex. (A)** Bars depict the differential connectivity with pPPA and aPPA exhibited by both MPA and CON in both hemispheres (image inset above depicts the ROIs used for analysis). In both hemispheres there is significantly greater differential connectivity with CON than MPA but this difference is markedly stronger in the right hemisphere (*t*-tests between MPA and CON in each hemisphere separately). **(B)** Bars depict the differential connectivity with cIPL and OPA exhibited by both MPA and CON in both hemispheres (image inset above depicts the ROIs used for analysis). In both hemispheres there is significantly greater differential connectivity with CON than MPA but this difference is stronger in the right hemisphere (*t*-tests between cIPL and OPA in each hemisphere separately; ^∗^*p* < 0.01, ^∗∗∗^*p* < 0.0001). Solid bars represent left hemisphere, open bars represent right hemisphere.

For the ventral surface we observed a significant main effect of Hemisphere [*F*_(1,47)_ = 21.53, *p* =< 0.0001], reflecting on average greater differential connectivity with aPPA and pPPA in the left compared to right hemisphere. The main effect of Seed was also significant [*F*_(1,47)_ = 64.51, *p* = 2.28^-10^], reflecting on average greater differential connectivity for CON than MPA. Further, there was a significant Hemisphere by Seed interaction [*F*_(1,47)_ = 59.73, *p* = 6.49^-10^], indicating a stronger difference between MPA and CON in the right than left hemisphere (**Figure 11A**).

For the lateral surface, there was no significant effect of Hemisphere [*F*_(1,47)_ = 2.52, *p* = 0.12]. The main effect of Seed was significant [*F*_(1,47)_ = 123.79, *p* = 9.14^-15^], reflecting on average greater differential connectivity with OPA and cIPL for CON over MPA. There was also a significant Hemisphere by Seed interaction [*F*_(1,47)_ = 4.99, *p* = 0.03], again indicating a stronger difference between MPA and CON in the right than left hemisphere (**Figure 11B**). Thus, within the scene-related regions, CON shows a relatively stronger association than MPA with aPPA and cIPL.

Overall, our functional connectivity data highlight (i) differential patterns of connectivity among scene-selective regions with MPA more strongly associated with PPA ventrally than OPA laterally, (ii) significant differential connectivity with aPPA over pPPA localized to a region of medial parietal cortex anterior of MPA in both hemispheres and iii) stronger association of the connectivity-defined medial parietal ROI (CON), compared to MPA, with regions often associated with the memory network, including the precuneus, orbitofrontal cortex, and the anterior temporal lobe (Ranganath and Ritchey, 2012).

### Scene-Selectivity and Retinotopy in MPA and the Connectivity-Defined Region

**FIGURE 12:**
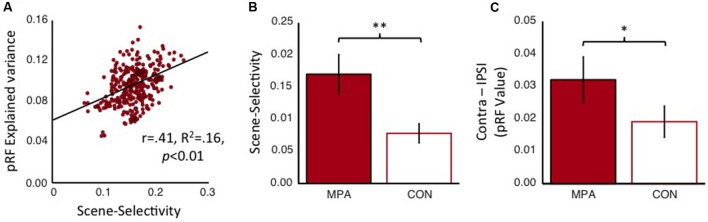
**Scene-selectivity and retinotopy within MPA and the connectivity-defined CON. (A)** The node-by-node correlation within MPA between scene-selectivity and explained variance of the pRF model (collapsed across hemispheres). These two functional properties are significantly positively correlated, suggesting that the same neural populations are capable of representing both scene-relevant visual features and retinal location. **(B)** Group-average scene-selectivity within the retinotopically defined MPA and CON, respectively. Although both regions exhibit evidence for scene-selectivity, this is significantly more pronounced within MPA. **(C)** Group-average contralateral biases (Contralateral *minus* Ipsilateral pRF value) within scene-selective MPA and CON, respectively. Again, despite both regions showing an overall contralateral bias, this bias is significantly more pronounced within MPA. ^∗^*p* < 0.05, ^∗∗^*p* < 0.01.

Finally, we compared these two regions within medial parietal cortex in terms of both scene-selectivity and retinotopy. Initially, we examined the relationship between these two functional properties within the MPA alone. Within our group-based MPA ROIs we calculated the correlation between each nodes scene-selectivity index and the explained variance of the pRF model (**Figure 12A**). This analysis revealed a significant positive correlation (*r* = 0.41, *R*^2^ = 0.16, *p* < 0.01), suggesting, that in general, nodes within MPA that show greater scene-selectivity also tend to show higher retinotopic sensitivity. These data provide no evidence for separate nodes that are selectively sensitive to either type of visual information (scene-selectivity and retinotopy) alone and suggest that both types of visual information are capable of being represented by the same underlying population of nodes. Second, we examined the scene-selectivity exhibited by both MPA and the CON. To avoid any circularity in this analysis our MPA ROI was redefined, not in terms of its scene-selectivity as before, but in terms of its retinotopic sensitivity. That is, we selected only nodes within the POS that exhibited significant retinotopic responses and then calculated the average scene-selectivity exhibited by both regions. Consistent with our original definition, MPA was found to exhibit significantly greater scene-selectivity than CON [*t*_(14)_ = 2.62, *p* < 0.01] (**Figure 12B**). Finally, we calculated the contralateral bias exhibited by both MPA (defined by its scene-selectivity) and CON. Again, MPA exhibited a significantly greater contralateral bias [*t*_(14)_ = 2.04, *p* < 0.05], although of note, CON also exhibited a numerical bias to the contralateral visual field (**Figure 12C**). These data support the conclusion that MPA is more sensitive than CON to both retinotopic location and visual scene information and suggest a gradient of visual responsiveness across the medial parietal surface.

## Discussion

In this study, we combined pRF and category-selectivity mapping with independently acquired resting-state analyses to explore scene and retinotopic processing in medial parietal cortex. We identify a region of scene-selectivity within the posterior and ventral banks of POS that we term the MPA. Our pRF data not only highlight strong spatial sensitivity within this region, but also, the presence of significant contralateral visual field biases, coupled with large pRFs [mean (se) 7.01° (0.52°)]. Our resting state analyses highlight differential patterns of connectivity between MPA and PPA over OPA. Moreover, a region directly anterior of MPA exhibited significant differential connectivity with anterior over posterior PPA divisions, and strong connectivity with cIPL, suggesting a posterior–anterior gradient exists within medial parietal cortex from retinotopic and scene-related representations more posteriorly to more abstract, navigationally relevant and potential mnemonic representations more anteriorly.

### Scene-Selectivity in Medial Parietal Cortex

Although originally labeled ‘RSC’ (Epstein, 2005), we, along with others (Nasr et al., 2011; Marchette et al., 2014), find the medial scene-selective region to be located largely within the ventral and posterior bank of POS and generally, spatially separate from both retrosplenial cortex proper (Morris et al., 2000) and the representations of the far periphery in V1 and V2d.

The precise boundaries of human retrosplenial cortex are actively debated in the literature, with conflicting opinions as to its spatial extent with respect to the cingulate gyrus (Maddock, 1999; Morris et al., 2000; Vogt et al., 2000; Maguire, 2001). Retrosplenial cortex corresponds to Brodmann areas 29/30, which encircle the posterior and ventral portion of the corpus callosum (Zilles and Palomero-Gallagher, 2001; Vann et al., 2009), and are in turn partially encircled by the posterior cingulate (BA 23 and 31; Maguire, 2001; Epstein, 2008). Although Brodmann regions have been converted to Talairach space for visualization on cortical surfaces, a number of inaccuracies in the mapping from Brodmann to Talairach have been reported (Vogt et al., 1995) making the accuracy of this mapping difficult to interpret. A detailed analysis of the anatomical location of retrosplenial cortex (Morris et al., 2000) suggests that, like in macaque, its dorsal component is buried within the callosal sulcus, with only the posteroventral portion extending onto the medial surface overlapping the isthmus of the cingulate gyrus (Morris et al., 2000; Maguire, 2001). This anatomical location is both slightly ventral of the tip of the calcarine sulcus and also anterior of the ventral portion of the POS (see Figure 2B in Morris et al., 2000; Figure 1A in Vann et al., 2009 and Shine et al., 2016).

Importantly, the location of scene-selectivity we report here is located largely within the ventral and posterior bank of the POS – it is not only dorsal of the anterior tip of the calcarine sulcus, but also, dorsal and posterior to the isthmus of the cingulate gyrus (Morris et al., 2000; Maguire, 2001), suggesting that it does not extend into retrosplenial cortex proper. A similar spatial separation (estimated at ∼1 cm) between retrosplenial cortex and medial scene-selectivity has been reported previously (Nasr et al., 2011). Such spatial separation from retrosplenial cortex suggests, as others have noted (Nasr et al., 2011; Marchette et al., 2014), that the continued use of ‘retrosplenial’ to refer to this medial scene-selective region may cause unnecessary confusion. We propose adopting a similar nomenclature to that used for PPA (Epstein and Kanwisher, 1998) and more recently OPA (Nasr et al., 2011; Dilks et al., 2013) and argue for the use of the MPA to refer to this visually defined scene-selective region of medial parietal cortex.

### Posterior to Anterior Gradient Within Medial Parietal Cortex

We demonstrate a posterior–anterior gradient across medial parietal cortex in terms of both retinotopic properties and functional connectivity (Supplementary Figure S3).

#### Retinotopy within Medial Parietal Cortex

Our pRF analyses demonstrate that MPA evidences strongly retinotopic voxels, which exhibit significant contralateral biases despite large receptive field sizes. Our analysis based solely on the strength of pRF responses highlighted a region within the ventral and posterior bank of POS that overlapped substantially with our MPA ROI in both hemispheres. In both cases, this region was spatially separate from representations of the far periphery in early visual cortex and from visual field maps present in dorsal POS (Pitzalis et al., 2006; Cardin et al., 2012; Pitzalis et al., 2015), suggesting that these responses do not simply reflect spatial blurring of retinotopy from either antecedent or dorsal retinotopic regions. If this region were simply an extension of retinotopy within V1 and V2d it would represent the very far periphery, which was not stimulated in our current pRF mapping paradigm. Importantly, the retinotopy we demonstrate here was only present within posterior and ventral portions of medial parietal cortex, showing a tight coupling to the location of scene-selective MPA.

#### Functional Connectivity within Medial Parietal Cortex

The stronger connectivity with aPPA compared with pPPA in medial parietal cortex anterior to scene-selective MPA is consistent with previous reports (Baldassano et al., 2013). However, whilst previous work emphasized some overlap between this region of differential connectivity and scene-selective regions, we find that the strongest differential connectivity with aPPA falls directly adjacent and anterior of MPA, with limited overlap in either hemisphere. The striking spatial adjacency between our scene- and retinotopically sensitive MPA, within the posterior banks of ventral POS, and the more anterior region of strong differential connectivity with aPPA, suggests a gradient in the representation of scene-related visual information within medial parietal cortex along a posterior–anterior axis. Indeed, our direct comparison of MPA vs. our CON demonstrates stronger associations between MPA and posterior visual and category-selective regions, which have been shown previously to be highly retinotopic (Sayres and Grill-Spector, 2008; Silson et al., 2015, 2016), whereas significant CON associations were found within the cIPL and precuneus, as well as anterior temporal, superior frontal, and orbitofrontal areas, respectively.

Our finding of a region anterior of scene-selective MPA that shows strong associations with regions likely involved in high-level spatial-, navigational- and memory-related processes is consistent with a number of recent reports (Marchette et al., 2014; Gilmore et al., 2016). For example, Marchette et al. (2014) utilized searchlight analyses to identify regions of the brain from which imagined facing direction could be decoded successfully. In the right hemisphere, this region was located within the anterior bank of ventral POS and in the left hemisphere this region was anterior and slightly dorsal, of both the anterior bank of POS and RSC (we term MPA; Marchette et al., 2014). Moreover, Gilmore et al. (2016) compared the cortical responses for remembered over imagined events, which highlighted, among other regions, ventral and anterior portions of POS. The proposed gradient within medial parietal cortex, based on the differential connectivity with anterior and posterior PPA divisions is also compatible with a very recent proposal for two scene processing networks derived from functional connectivity data (Baldassano et al., 2016). In this framework, the first network comprises OPA and pPPA and is thought to be more related to processing visual features, whereas the second network consists of cIPL, aPPA, and the RSC (we term MPA). Taking this framework into account, our data suggest that the visually selective MPA is likely associated with the first network, whereas the more anterior region in precuneus (CON) is more associated with the second network.

### Differential Functional Connectivity among Scene-Selective Regions

The disproportionately strong relationship between MPA and PPA reported here is consistent with previous resting-state connectivity results (Kim et al., 2015), demonstrating that PPA exhibits the strongest connectivity when seeding RSC (we term MPA) in healthy controls, but not a patient with topographical disorientation (Kim et al., 2015). Moreover, the relatively weak association we observe between MPA and OPA is similar to the weak association reported previously for RSC (MPA) and other category-selective regions including LOC and FFA (Kim et al., 2015). These data suggest that MPA is more closely associated with PPA on the ventral surface than with OPA on the lateral surface, despite sharing a common selectivity profile.

### Retinotopy within MPA

Although significant responses to contralaterally (over ipsilaterally) presented scenes have been reported previously within this region (MacEvoy and Epstein, 2007), a systematic evaluation of pRFs has hitherto not been conducted. Indeed, the suggestion that this region is largely insensitive to visual field position and may contain receptive fields that span the vertical meridian were made largely on the basis of larger ipsilateral responses in this region relative to either OPA or PPA and fMRI adaptation that was tolerant to positional changes (MacEvoy and Epstein, 2007). Here, we demonstrate that activity within MPA is modulated systematically by visual field position and that receptive fields of MPA voxels are not only more abundant in the contralateral visual field, but also, that the representations are largely confined therein - although some representation of the ipsilateral visual field is expected given the large size of MPA pRF’s and the assumption of circular RFs in our model (Silson et al., 2015). A previous study (Huang and Sereno, 2013), reported a representation of the far periphery, coupled with an upper field representation within a medial region similar in location to MPA. We too demonstrate peripheral representations within MPA, but do not observe a consistent upper visual bias across either our pRF or event-related analyses. Differences in the methods for both localization of MPA and retinotopy could underlie these discrepancies. For instance, the previous report identified their medial parietal ROI using a mental navigation task and employed a retinotopic mapping paradigm whereby coherent scene clips from a television series were revealed gradually (Huang and Sereno, 2013).

Clear and orderly progressions of the visual field within MPA were not visible consistently in our data, despite the strong retinotopic sensitivity shown by MPA as a whole. MPA’s large receptive fields may prohibit accurate estimates of polar angle in this region under the current pRF paradigm as voxels will respond to a number of visual field positions. Additionally, we do not observe clear clustering within MPA for different quadrants of the visual field (e.g., upper or lower). Again, the large receptive fields within MPA could underpin this. Importantly, however, these data cannot rule out the possibility that a map(s) of the visual field within MPA could be delineated with more sophisticated mapping paradigms at higher resolution. MPA exhibits large pRF sizes are suggestive of more global computations allowing neurons within MPA to pool visual scene information across large areas of the contralateral visual field. The large size of MPA pRF’s likely underpin the more complete hemifield representation exhibited by MPA, compared to the largely quadrant representations within OPA (contralateral lower) and PPA (contralateral upper), respectively (Silson et al., 2015). Although currently not known, it is possible that MPA receives input from dorsal visual field maps within POS (V6A, V6Ad, and V6Av), which all represent the contralateral hemifield, rather than direct input from OPA, which represents predominantly the contralateral lower field (Silson et al., 2015, 2016). A largely complete hemifield representation within MPA suggests further that MPA plays a role in mediating scene-relevant visual information between OPA on the lateral surface (lower visual field biased) and PPA on the ventral surface (upper visual field biased), whose visual field representations only overlap at the fovea (Silson et al., 2015). Finally, our comparison of scene-selectivity and retinotopy within MPA demonstrates a significant positive correlation suggesting that the same underlying neural populations are capable of encoding both scene-relevant visual features and their retinotopic location, rather than being two separate neural populations within MPA.

## Conclusion

Together, our data highlight that scene-selective MPA, in the posterior and ventral banks of POS, evidences strongly retinotopic voxels, contains large receptive fields and represents predominantly the contralateral visual field. MPA is also differentially connected to ventral (PPA) over lateral (OPA) scene-selective regions. Dividing PPA into posterior and anterior portions revealed a region of medial parietal cortex directly adjacent and anterior to MPA that exhibited highly significant aPPA associations. The spatial adjacency within medial parietal cortex, exhibited by this region and our scene- and retinotopically sensitive MPA, suggests that scene-related visual information undergoes a transformation from strongly retinotopic and visually grounded in posterior and ventral portions of POS, to more abstract, navigationally- and memory-relevant more anteriorly.

## Author Contributions

ES jointly designed the study, collected and analyzed the retinotopic, event-related and functional localizer fMRI data, and wrote the manuscript. AS collected and analyzed the resting-state functional connectivity data and wrote the manuscript. CB jointly designed the study and wrote the manuscript.

## Conflict of Interest Statement

The authors declare that the research was conducted in the absence of any commercial or financial relationships that could be construed as a potential conflict of interest.

## Footnotes

1 http://afni.nimh.nih.gov/afni

2 http://surfer.nmr.mgh.harvard.edu/
