# Supplementary figures and images for: Scene-Selectivity and Retinotopy in Medial Parietal Cortex
Source: Front Hum Neurosci. 2016 Aug 18;10:412. doi: 10.3389/fnhum.2016.00412 (PMC4988988; doi:10.3389/fnhum.2016.00412)

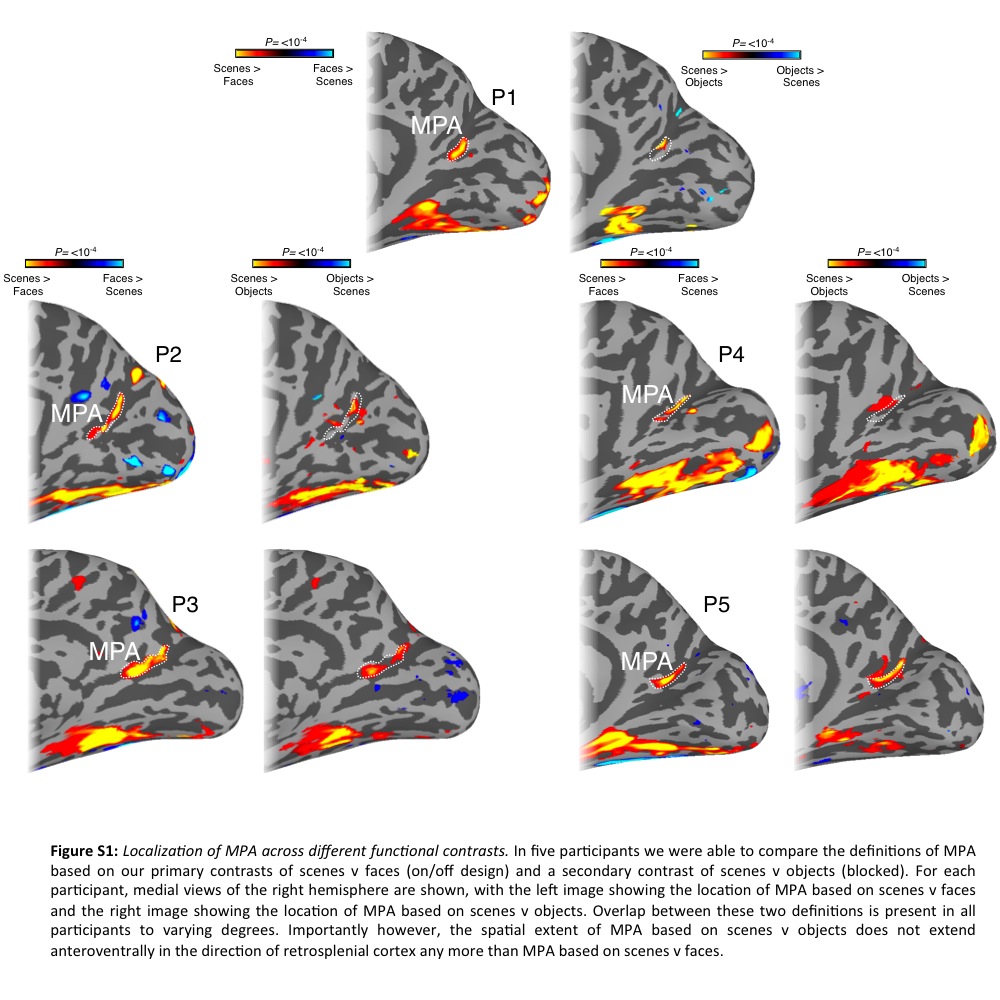

Supplement: Supplementary file 1 [file Image_1.JPEG]

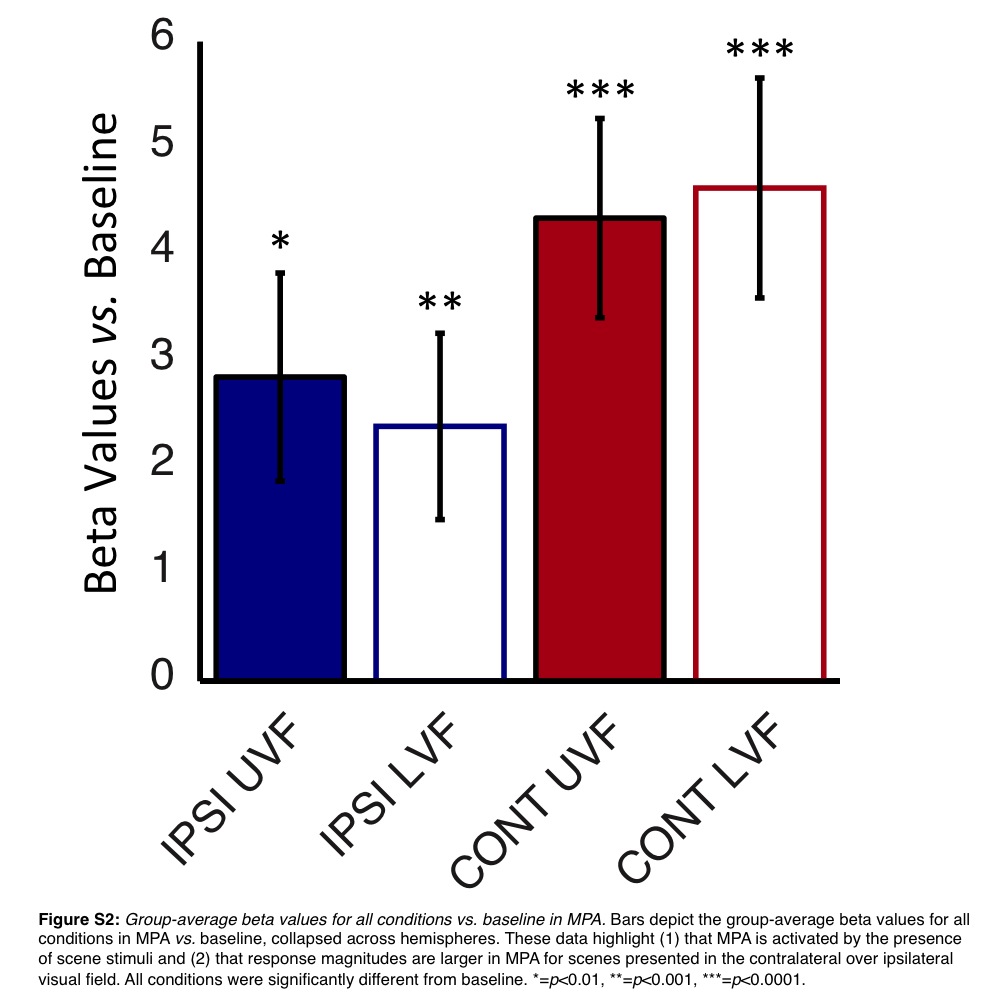

Supplement: Supplementary file 2 [file Image_2.JPEG]

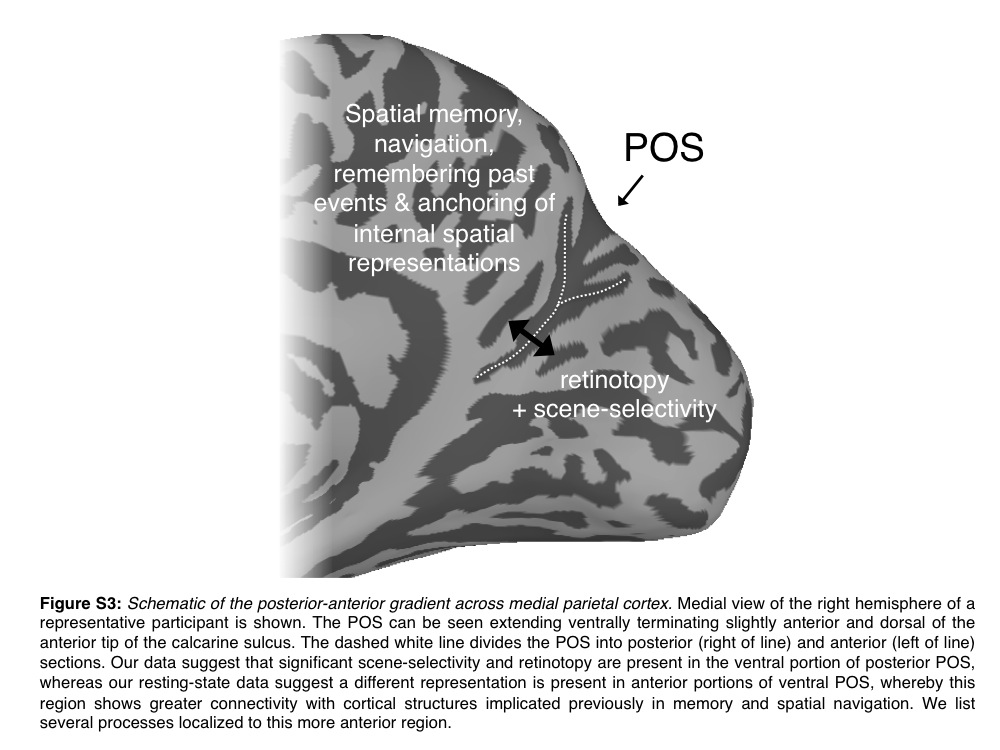

Supplement: Supplementary file 3 [file Image_3.JPEG]
